# Disaster management: a study on knowledge, attitude and practice of emergency nurse and community health nurse

**DOI:** 10.1186/1471-2458-12-S2-A3

**Published:** 2012-11-27

**Authors:** Nurul’Ain Ahayalimudin, Aniza Ismail, Ismail Mohd Saiboon

**Affiliations:** 1 Kulliyyah of Nursing, International Islamic University Malaysia, Jalan Hospital Campus, 25100 Kuantan, Malaysia; 2 Department of Community Health, Universiti Kebangsaan Malaysia Medical Centre, Jalan Yaacob Latif, Bandar Tun Razak, 56000 Kuala Lumpur, Malaysia; 3 Department of Emergency Medicine, Universiti Kebangsaan Malaysia Medical Centre, Jalan Yaacob Latif, Bandar Tun Razak, 56000 Kuala Lumpur, Malaysia

## Background

Disasters are unpredictable events that kill and affect people, demolish properties and disrupt environment. During such events, nurses play a vital role in dealing with the victims. It is therefore crucial for nurses to be prepared in facing the aftermath of disasters. The aim of this study was to determine knowledge, attitude and practice of emergency nurse and community health nurse towards disaster management.

## Materials and methods

This was a cross-sectional study conducted in emergency departments and health clinics in Selangor, one of the states in Malaysia. Questionnaire forms eliciting information about knowledge, attitude and practice towards disaster management were randomly distributed to 468 nurses working at the aforementioned clinics. This survey was conducted from October to November 2011 and yielded a response rate of 84.6 per cent.

## Results

Both groups of nurses had similar inadequate knowledge but portrayed positive attitude towards disaster management. They differ in terms of practice whereby 56.1% of emergency nurses reported having had adequate practice compared to 30.7% of the community health nurses (chi-squared test, P<0.001). Emergency nurses who have been involved in disaster response are more likely to report adequate practice (P<0.01, AOR=4.008, 95% CI=1.691-9.504) while those who attended disaster-related education/training are more likely to have adequate knowledge (P<0.05, AOR=3.807, 95% CI=1.584-9.153) and practice (P=0.001, AOR=4.145, 95% CI=1.804- 9.525). Attending disaster-related education/training is seen to be a predictor to adequate knowledge (P<0.001, AOR=3.511, 95% CI=2.097-5.881) and practice (P<0.001, AOR=4.080, 95% CI=2.326-7.156), and portraying positive attitude (p<0.05, AOR=2.042, 95% CI=1.025-4.069) among community health nurse. Nurses’ workplace (represent type of nurse) is identified as a predictor for the adequacy of practice (P<0.001, AOR=2.345, 95% CI=1.474-3.730).

## Conclusions

Adequacy of knowledge and practice, and portraying positive attitude is driven by being involved in disaster response and attending disaster-related education. It is therefore paramount for health administrators to conduct disaster-related education/training for front-liners such as emergency and community health nurses to improve their knowledge and practice towards disaster management.

